# Improving energy balance of wastewater treatment plants using pre-treated coffee waste as a co-substrate in anaerobic digestion process

**DOI:** 10.1007/s11356-025-37346-8

**Published:** 2026-01-07

**Authors:** Aleksandra Szaja, Agnieszka Montusiewicz, Rafał Panek, Iwona Musielewicz, Magdalena Lebiocka

**Affiliations:** 1 Department of Biomass and Waste Conversion Into Biofuels, Faculty of Environmental Engineering and Energy, Lublin University of Technology, Nadbystrzycka St. 40B, Lublin, 20-618 Poland; 2 Department of Building Materials Engineering and Geoengineering, Faculty of Civil Engineering and Architecture, Lublin University of Technology, Nadbystrzycka 40, Lublin, 20-618 Poland; 3 Environmental Analysis Laboratory, Faculty of Environmental Engineering and Energy, Lublin University of Technology, Nadbystrzycka St. 40B, Lublin, 20-618 Poland

**Keywords:** Coffee spent grains, Methane production, Energy efficiency, Kinetics, Fourier transform infrared spectroscopy with attenuated total reflectance accessory, Scanning electron microscopy

## Abstract

In this study, the strategy for improving energy balance of wastewater treatment plants using pre-treated coffee waste represented by coffee spent grains (SCG) as a co-substrate to sewage sludge (SS) was proposed. Hydrodynamic cavitation at a pressure of 5 bar with a duration of 20 and 30 min was chosen as a pre-treatment method. The anaerobic digestion was conducted in a batch system under mesophilic conditions; the substrate-to-inoculum ratio was 0.69–0.71. Two co-digestion series with cavitated SCG were provided, differing in terms of caffeine content. Moreover, the co-digestion series supplied with raw SCG and mono-digestion of SS were conducted as reference experiments. The obtained results indicated that, compared to SS mono-digestion, a significant yielding growth by 12% was found in the series supplied by a mixture of SS and SCG with the highest content of caffeine cavitated for 30 min. Therein, the methane yield was 462.3 ± 21.3 mLCH_4_/gVS accompanied by improved methane production rate by 9% and shortening of the lag phase. The evaluation of energy balance with the significant energy profits of 39% was found. Moreover, the results of the economic analysis indicate that only in this case the cost-effectiveness of the technology was achieved.

## Introduction

Municipal wastewater treatment plants (WWTPs) are recognised as a potential source of renewable energy and raw materials. On the other hand, many facilities are still considered as highly energy consuming and emitting (Huang et al. 2022). Generally, the average energy consumption for WWTPs varied over a wide range from 0.243 to 0.53 kWh/m^3^, depending on the adopted treatment line, the type and concentration of pollutants, as well as quality standards for the treated wastewater (Tsalas et al. 2024). This high energy consumption leads to significant emissions of greenhouse gases; the WWTPs located in the Baltic Sea region’s energy consumption was responsible for over 30% of the total emissions (Maktabifard et al. 2022).

Therefore, the attention of scientists and WWTP operators has been focused on achieving energy self-sufficiency and even carbon neutrality (Zhang et al. 2024). One of the possibilities to achieve those goals is to implement the anaerobic co-digestion (AcoD) strategy. The previous studies from existing facilities demonstrated the great potential of this strategy. Many WWTPs, e.g. Zurich Wedholzli in Switzerland, Point Loma in the USA, Grevesmuhlen in Germany, and Sheboygan Regional in the USA, achieved energy neutrality or even became net energy positive (Nguyen et al. 2021; Maktabifard et al. 2023).

Despite many benefits, such as increased methane production, improved process stability and quality of digestate, the application of AcoD is still a technological challenge (Akca et al. 2024; Masłoń et al. 2024). Those difficulties are mainly related to selecting the appropriate component to balance the deficiencies of the main substrate and establishing optimal process conditions. Other factors that should be considered are seasonality and availability on the local market of co-substrates. Previous studies have confirmed that the AcoD of SS and various waste generated by the food industry proceeds synergistically, resulting in enhanced methane production (Chen et al. 2024; Thakur et al. 2024). The most commonly applied co-substrates at municipal WWTPs are the residues obtained from meat processing plants, fruit and vegetable processing companies, slaughterhouses and dairies, as well as facilities producing fats and oils (Masłoń et al. 2020; Chow et al. 2020; González et al. 2022). For instance, at Iława WWTP, the introduction of poultry processing waste led to significant improvement in biogas production, allowing in this way to cover the energy demand by 98.2%, on average (Masłoń et al. 2020). At the Treviso WWTP, the use of the organic fraction of municipal solid waste led to achieving electric energy coverage of 49% (Moretto et al. 2020). In turn, the introduction of slaughterhouse waste to existing digesters located at Foligno WWTP promoted biogas production and hence increased recoverable energy (Salehiyoun et al. 2020).

For example, the use of oil wastes led to 4-times increased biogas production, resulting in an energy profit of 113%. Similarly, using grease trap water as a co-substrate led to a biogas production increase of up to 209% (Neczaj and Grosser 2018). The introduction of carbonated soft drinks as a co-substrate led to an increase in biogas production by 191% (Wickham et al. 2018). Significant enchantment was also achieved in the case of using glycerol, therein 81% impairment in biogas production was achieved (Nghiem et al. 2014).

One of the potential co-substrate generated globally is spent coffee ground (SCG) as the main solid by-product generated during the coffee-making process. Currently, coffee is one of the most frequently consumed beverages in the world, reaching the level of 10.1 billion kg in 2020 (Montemurro et al. 2024) and contributing to the generation of significant amounts of waste that may pose a threat to the environment.

Previous studies indicated that SCG has a potential for bio-energy production, mainly due to high organic content. However, its mono-digestion is ineffective, even despite supplementation of micronutrients or ensuring alkalinity (Kim et al. 2017a, b). The presence of hemicellulose and lignin, two main components of SCG, causes that it is resistant to enzymatic hydrolysis (Girotto et al. 2018). A potential solution might be its pre-treatment prior to anaerobic digestion. Various methods have been applied, e.g. acid thermal pretreatment, alkaline (Girotto et al. 2018), oil extraction (Atelge et al. 2020) or mild-temperature thermo-alkaline pretreatment (Kim et al. 2017a, b). However, all mentioned methods are related to reagent dosing and financial outlays. More favourable results were found when SCG was co-digested with food waste, Ulva, waste activated sludge, and whey (Kim et al. 2017a, b) as well as pig manure (Orfanoudaki et al. 2020). However, new cost-effective solutions are still being sought, allowing for releasing the energy potential of this waste. Particularly, its application in co-digestion with sewage sludge should be further developed due to the untapped potential of the digesters applied at WWTPs. Another aspect that should be investigated refers to the influence of caffeine on the methane production that is not fully recognised. Depending on the caffeine concentration, its influence might be stimulating, while at increased concentration, it can have an inhibitory effect on the AD process (Chen et al. 2018; Prabhudessai et al. 2009).

In this work, a novel strategy for improving the energy balance of WWTPs by using pre-treated coffee waste as a co-substrate to sewage sludge (SS) was proposed. As the pre-treatment method, a hydrodynamic cavitation (HC) was chosen. Previous studies demonstrated that HC might be applied as a profitable technique for improving the biodegradability of various wastes, including lignocellulosic biomass (Szaja et al. 2024; Dębowski et al. 2024). In relation to other recognised pre-treatment methods, HC is characterised by easy operation and application in full-scale facilities, low operating cost and high energy efficiency. Moreover, it can be easily combined with other available technologies (Wang et al. 2021). The influence of pre-treated SCG on the AcoD process was examined on the basis of volatile solids (VS) removal, process stability, methane production and its kinetics. The effect of caffeine on anaerobic digestion of SS was also evaluated. To illustrate the changes occurring during the anaerobic conversion, Fourier transform infrared spectroscopy with attenuated total reflectance accessory (FT-IR/ATR) as well as scanning electron microscopy (SEM) was applied. In order to estimate possible energy gains, an energy balance was performed. Such multi-faceted research involving SCG has not been conducted thus far. Importantly, the proposed technology will allow for effective management of SCG with energy recovery, contributing to the improvement of the energy efficiency of existing WWTPs.

## Materials and methods

### Operational set-up and laboratory installations

The anaerobic digestion experiment was performed in a batch mode under mesophilic conditions (37 °C), involving laboratory-scale equipment BioReactor Simulator BRS III provided by BPC instruments. In this study, two equal units were applied. Each unit included 6 reactors with a capacity of 2 L. All reactors were equipped with mechanical agitation. A water bath was utilised for maintaining the adopted temperature conditions. The volume of biogas was recorded using a flow cell unit working on the principle of buoyancy and liquid displacement. In this part of the study, four experimental series (S0–S3) were provided, differing in feedstock composition (Table 1). All reactors were fed by 1.4 L of inoculum as well as 0.4 L of SS. In order to ensure anaerobic conditions, the gas space was flushed every time, primarily with nitrogen.

**Table 1:** The operational conditions in anaerobic digestion experiment

Series	Feedstock composition	VS load in the feedstock g/kg
S0-mono-digestion	0.4 SS	13.16 ± 2.1
S1-co-digestion	0.4 SS + 1.5 g non-cavitated SCG	13.86 ± 1.1
S2-co-digestion	0.4 SS + 1.5 g SCG cavitated for 20 min	13.30 ± 0.7
S3-co-digestion	0.4 SS + 1.5 g SCG cavitated for 30 min	13.33 ± 1.2

SCG was pre-treated using HC. The pretreatment was conducted involving a laboratory device comprising a cavitation reactor, circulation tank, pump with inverter, piezoelectric pressure gauges and electromagnetic flow metre. The detailed characteristics of the system are given in the authors’ previous work devoted to multi-criteria decision support in terms of performing HC as coffee waste pre-treatment (Szaja et al. 2024). To conduct HC, SCG was suspended in a carrier; for this purpose, municipal wastewater after primary treatment was applied. Raw SCG in the amount of 950 g was added to 30 L of MW. HC occurred using an inducer in the form of a 64-mm plate with a central hole of 3 mm inlet and 10 mm outlet diameters at 5 bar inlet pressure and a duration of 20 and 30 min for S2 and S3, respectively, that corresponds with passing mixture through the cavitation zone 14.6 and 21.9 times. The cavitation number was 0.067. Multi-criteria decision support indicated that the parameters listed allowed for significant biodegradability improvement, with simultaneous low energy usage. Nevertheless, caffeine was released at 30 min; however, its concentration was relatively low. In turn, for the time of 20 min, there was no release of this compound during HC. The adoption of these two times will allow determining the impact of caffeine on the efficiency of the anaerobic digestion process.

Two co-digestion series with hydrodynamically cavitated SCG were carried out, differing in terms of caffeine presence (S2, S3). In the first one, within HC of SCG, the caffeine was not detected (S2), whereas in the second one, the release of caffeine was observed (S3). To assess the impact of HC on the effectiveness of SCG and SS co-digestion, the reactor supplied with non-cavitated SCG (S1) was established as a reference one. Additionally, to perform energy balance and economic analyses, the control series (S0) where mono-digestion of SS was conducted was provided. In all co-digestion experiments, SCG was provided to the reactors in equal amounts of 1.5 g DM to 0.4 L of SS. Each experimental series lasted 21 days, and it was repeated three times. The substrate-to-inoculum ratio (S/I) ranged from 0.69 to 0.71. Feedstock composition and the average data of volatile solids (VS) load are presented in Table 1. The adopted S/I ratio, as well as doses of SCG, was established to ensure the most favourable conditions for methane production and stable process performance. As it was previously mentioned, SCG cavitated for 20 min and 30 min was chosen because of significantly improving the biodegradability of the SCG. Both samples differed in caffeine content. As it can be seen in Table 1, the introduction of co-substrates led to an increase in the VS load in feedstock; the major growth was found in S1 supplied with raw SCG, while the minor effect was observed in SCG cavitated for 20 and 30 min, respectively. This finding was related to the destruction of complex organic compounds within HC.

### Characteristics of applied substrates

In this research, SS constituted the primary substrate. The sample was collected from the Hajdów mechanical-biological WWTP in Lublin (Poland). It constituted primary and excess sludge mixed at the volumetric ratio of 60:40 v/v. From the same facility, an inoculum for batch experiments was collected from the mesophilic digester. The inoculum was characterised by the following parameters: total solids (TS) and volatile solids (VS) of 23.47 ± 0.15 and 13.92 ± 0.21 g/kg, respectively, and a pH of 7.34 ± 0.05. SCG was taken from coffee machines on the Lublin University of Technology campus (Poland). The carrier to perform HC, e.g. mechanically treated municipal wastewater, originated from the same WWTP. Table 2 shows the characteristics of the utilised substrates, including chemical oxygen demand (COD), soluble fraction of chemical oxygen demand (sCOD), TS, VS and pH, as well as phenol and caffeine concentration.

**Table 2:** Characteristics of all substrates applied in the anaerobic digestion experiment (including average values and standard deviation)

Description	COD mg/L	sCOD mg/L	TS g/kg	VS g/kg	Phenols mg/L	pH	Caffeine ppb
Wastewater	562 ± 12.3	487 ± 9.8	4.3 ± 0.8	3.52 ± 0.9	3.15 ± 0.5	7.42 ± 0.05	nd
Non-cavitated SCG	9820 ± 189	1310 ± 25	9.21 ± 2.1	8.11 ± 0.7	17.60 ± 1.7	7.38 ± 0.07	nd
Cavitated SCG for 20 min	6597 ± 155	1720 ± 45	8.20 ± 1.7	7.41 ± 0.5	22.70 ± 2.5	7.83 ± 0.08	nd
Cavitated SCG for 30 min	6928 ± 164	1828.3 ± 36	7.94 ± 0.8	7.08 ± 0.5	25.10 ± 3.1	7.94 ± 0.1	510 ± 32
SS	47,900 ± 245	2384 ± 77	45.49 ± 4.4	33.55 ± 2.7	3.45 ± 0.05	5.99 ± 0.04	nd

### Analytical methods

#### The physicochemical analyses

In all substrates, the content of COD, sCOD, TS and VS, content of phenols and caffeine, and pH levels were analysed. The process efficiency was evaluated using removals of organic compounds, including VS, TS, COD and sCOD. In turn, to examine the process stability, the concentrations of volatile fatty acids (VFA), alkalinity (ALK) and pH, as well as VFA/ALK ratio, were controlled in the feedstock and digestate of the reactors. Most of these indicators were marked using a DR 3900 spectrophotometer and standard cuvette tests (Hach Lange). In the case of TS and VS, the process described in APHA 2012 was applied. The pH value was read using a CP-411 pH-metre (Elmetron).

The composition of the biogas (CH_4_, CO_2_) was controlled involving a ThermoTrace GC-Ultra gas chromatograph (Thermo Fisher Scientific). The conductivity detector with divinylbenzene (DVB) packed columns (RTQ-Bond) was applied for the analyses. The temperature of the injector was 50 °C; in turn, for the detector, it was established at 100 °C. The employed carrier gas was helium with a flow rate of 1.5 cm^3^/min.

The caffeine content was determined chromatographically as well, the temperature of the injector was maintained at 270 °C, and the applied column was Equity5MS Supelco. The temperature programme consisted of 40 °C for 2 min, then increased 8 °C/min to 150 °C and 10 °C/min to 270 °C.

##### FT-IR/ATR

The analysis was carried out using a THERMO i50Nicolet spectrometer. The determinations were performed three times for every sample, and the result was averaged; atmospheric correction was applied, and the spectra were not subjected to any processing. A diamond attachment was used; the resolution was 4 with the number of scans 1064.

##### SEM

An ultra-high-resolution analytical FIB-SEM Scios 2 LoVac (Thermo Fisher Scientific) with an UltraDry Premium EDS chemical composition analyser was used to analyse the surface morphology of the samples. The microscope included a Schottky cathode as well as an NICol UHR electron column in nonimmersion mode. Dried specimens were mounted on aluminium holders with carbon tape, without sputtering, to enable high-magnification imaging without additional conductive layers. Surface studies were carried out in high vacuum using the Thermo Scientific Trinity detection system in OptiPlan mode, allowing simultaneous angular image registration and energy-selective SE and BSE imaging at 2–5 keV accelerating voltage and 0.1–0.2 nA beam current.

#### Kinetic evaluation

Kinetic evaluation was performed using two models, i.e. Modified Gompertz (1) and Logistic growth (2) were selected to perform. Each of them showed the optimal fit to the experimental data, that is confirmed by root-mean-square error (RMSE) values (3) below 10. Additionally, for selected models, the high values of determination coefficient (*R*^2^) exceeding 0.99 were achieved, confirming that these models are suitable for simulating the kinetic parameters of methane production.

1 $$P(t)={P}_{m}\bullet exp\left(-exp\left(\frac{{R}_{m}\bullet e}{{P}_{m}}exp\left(\lambda -t\right)+1\right)\right)$$

2 $$P(t)=\frac{{P}_{m}}{\left(1+ \mathrm{exp}(4\bullet {R}_{m}\bullet \left(\frac{\lambda -t}{{P}_{m}}\right)+2\right)}$$

3 $$\mathrm{RMSE}=\sqrt{\frac{{({P}_{exp}-{P}_{mod})}^{2}}{n}}$$

where *P*_*m*_ is the methane production (mL CH_4_/gVS), *R*_*m*_ is the maximum methane production rate (mL CH_4_/g VS d), *P(t)* is the cumulative methane production (mL CH_4_/gVS), *λ* is the lag phase (d), *k* is the rate constant (1/d), *e* is a constant (2.71828), *P*_*exp*_ is the methane production from the experiment (mL CH_4_/gVS), *P*_*mod*_ is the methane production from kinetic evaluation (mL CH_4_/gVS), and *n* is the number of measurements.

Both GPR (biogas production rate) and MPR (methane production rate) were evaluated on the basis of the following formula:

4 $$GPR/MPR=\frac{BP/P}{t}$$

where BP/P is the cumulative biogas/methane production (mL/gVS) and *t* is the duration of the experiment (*d*).

### Energy balance

On the basis of experimental data with regard to methane production, an energy assessment of the proposed technology was conducted to determine the energy gains and hence the profitability of the proposed solution. This evaluation is detailed in authors’ prior studies (Szaja and Montusiewicz 2019).

The evaluation was carried out for a full-scale mesophilic digester (35 °C) operating with the active volume of 2500 m^3^, an average feedstock flow rate of 125 m^3^/d and HRT of 20 d. The calculations were performed for unfavourable conditions, e.g. the winter period with an outdoor temperature of −20 °C (Polish conditions), and SS temperature of 8 °C. The feedstock temperature was kept at 9 °C; the contribution of the cavitated mixture caused an increased temperature. The energy demand to perform one HC was maintained at 0.82 and 1.23 MJ for 20 and 30 min, respectively.

### Statistical analysis

Statistical analysis was performed using Statsoft Statistica software (v 13). Analysis of variance (ANOVA) (Shapiro-Wilk’s, Levene’s and Tukey’s tests) was chosen to assess the differences between results. Statistical significance was identified at *p* < 0.05. Kinetic constants were evaluated involving a nonlinear regression method. The relationships between the results were indicated using the Pearson’s correlation coefficient (*R*) and determination coefficient (*R*²). 

## Results and discussion

### Process performance

**Fig. 1:**
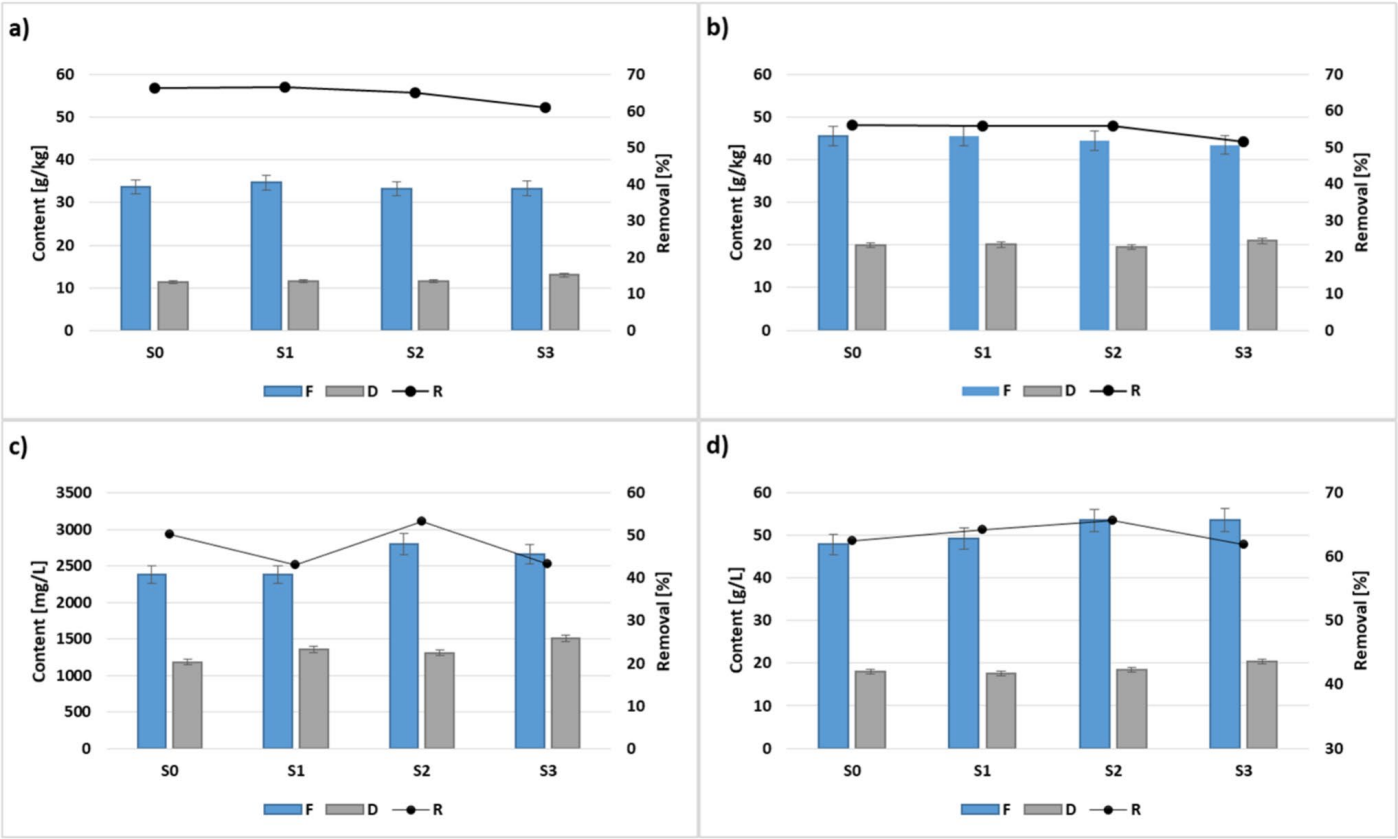
Content of **a** VS, **b** TS, **c** sCOD and **d** COD in feedstock (*F*) and digestate (*D*) and related removal efficiencies (*R*) (avg. ± SD, *p* < 0.05)

The process performance was evaluated on the basis of organic compounds removal with regard to VS, TS, sCOD and COD, respectively. As it is shown in Fig. 1a, VS removal in S0 and S1 was on a comparable level of 66%. In the presence of cavitated mixtures, a deterioration of this parameter was achieved. However, as compared to reference experiments (S0 and S1), a significant drop of 8% in VS removal was only observed in the case of S3, supplied by cavitated SGC by 30 min, where the caffeine was detected. A similar trend was observed for TS removal; therein, the average values varied between 51.7% and 56.1%, while the lowest value was achieved in S3. As is presented in Fig. 1c and d, the contribution of both cavitated mixtures to feedstock resulted in an improvement of both sCOD and COD as compared to both S0 and S1. However, the significant differences were found only for sCOD. This fact was related to the solubilisation effect occurring within HC, which is the result of the decomposition of complex structures in SCG (Garlicka and Żubrowska-Sudoł 2020; Wang et al. 2021; Bimestre et al. 2022). For sCOD removal (Fig. 1c), only in the case of S2 supplied by cavitated SCG by 20 min, the enhanced sCOD removal by 6% was achieved, as compared to S0, indicating its effective use by AD microbes. In other co-digestion series (S1 and S3), a significant drop of over 14% of this parameter was found. In this case, the soluble organic fraction is not fully metabolised by AD microbes. For COD removal (Fig. 1d), as compared to S0 in almost all co-digestion series, minor improvements were found. The exception was S3, where a slight reduction of this indicator was achieved. The obtained results indicated that in S3 supplied by cavitated SCG by 30 min, the AD process is characterised by lower efficiency as compared to SS mono-digestion and co-digestion with non-cavitated SCG. This may be due to the significant caffeine content in the feedstock of this reactor (Table 3). Previous studies have shown that caffeine is degraded by methanogens. However, its highly efficient degradation needs a long-term anaerobic process. For caffeine, the main rate-limiting step within its degradation is converting hydrolysis products to VFAs (Chen et al. 2018).

**Table 3:** The stability parameters and phenols as well as caffeine content in feedstock (*F*) and digestate (*D*) (average values and standard deviation are given)

Parameter	Unit		S0	S1	S2	S3
pH	-	F	5.99 ± 0.1	5.56 ± 0.15	5.41 ± 0.13	5.62 ± 0.1
		D	7.39 ± 0.11	7.43 ± 0.05	7.43 ± 0.1	7.43 ± 0.07
ALK	mg/L	F	794 ± 7.4	777 ± 5.3	896 ± 2.4	796 ± 5.5
		D	3970.5 ± 10.1	4086 ± 23.4	4138 ± 25.1	4179.5 ± 15.3
VFA	mg/L	F	647 ± 7.6	759 ± 8.8	843 ± 11.3	804 ± 12.3
		D	178.5 ± 10.1	205 ± 14.2	206 ± 5.7	216 ± 6.6
Phenols	mg/L	F	3.74 ± 0.05	6.54 ± 0.1	21.9 ± 1.3	18.23 ± 2.1
		D	2.96 ± 0.7	8.15 ± 1.1	8.25 ± 1.3	8.88 ± 0.7
Caffeine	ppb	F	nd	280.7 ± 17.1	287.7 ± 10.3	583.6 ± 21.5
		D	nd	nd	nd	nd

The process stability was evaluated on the basis of pH, alkalinity and VFA contents as well as VFA/ALK ratio. In all co-digestion series, the introduction of SCG to feedstock resulted in a significant drop in the pH value, as compared to control (S0). Moreover, relatively low pH values were found in all feedstocks. Generally, the value of pH 5.5 is considered a necessary level to initiate methanogenesis. Additionally, previous studies indicated a low pH value of SCG as a possibility of AD process failure (Girotto et al. 2018). However, in all reactors, the pH in digestate was established at a comparable level, adequate for stable process performance. The recommended value for efficient methane production should be varied between pH 6.8 and 8.5 (Mir et al. 2016). Moreover, in the presence of SCG, a significant increase of 24–30% in VFA content in feedstock was achieved. The same trend occurred with regard to digestate. Significant increases were found in all co-digestion series. However, it should be noted that relatively low values were observed in all reactors, thus confirming stable process performance. Inhibition occurs at VFA concentrations above 2000 mg/L (Siegert and Banks, 2005).

Another crucial parameter for effective AD is alkalinity, which is responsible for ensuring adequate buffer capacity and increased resistance to pH changes. The values between 1500 and 5000 mg/L are recognised as optimal for the AD process (Gerardi 2003; Wu et al. 2019). Only in the case of S2 supplied by cavitated SCG for 20 min, a significant improvement of 8% in the ALK value was observed. In other cases, comparable concentrations to the control reactor were achieved. Importantly, the VFA/ALK ratio in all series was established at the level indicating well-operated digesters (below 0.30). In the presented study, this ratio varied between 0.045 and 0.052, while the lowest value was observed in S0 (control reactor).

Another adverse effect of adding SCG is the significant increase in phenols and caffeine contents in the feedstocks. As it is shown in Table 4, major growths might be found in both cavitated SCG mixtures (S2 and S3) as a result of the transformations via HC. Both compounds are recognised as inhibitors of the AD process. The previous studies indicated that caffeine might inhibit the growth of several bacterial species (Isla et al. 2013). However, this effect depends mainly on its concentration. Values above 2500 mg/L might be considered as the inhibitory level, while the process breaks down when exceeding the value 4000 mg/L (Chen et al. 2018). In co-digestion systems, the negative effect of this alkaloid might be found. In a study conducted by Flisberg (2016), the addition of coffee grounds to algal biomass and sewage sludge resulted in a reduction in biogas production. A similar effect was achieved in the AD of food waste; in this case, the reduction of the caffeine content from 150 to 100 ppm enhanced biogas production over 20% (Prabhudessai et al. 2009). Importantly, in this study, despite the substantial content of caffeine, a significant increase in methane production was found, in particular in S3 (Table 3).

The increased content of phenols in S2 and S3 is related to the applied HC. Previous studies demonstrated that the application of various pre-treatments to lignocellulosic biomass resulted in the release of phenols. At high concentrations, phenolic compounds can inhibit the degradation of organic matter and reduce methane production. It is recognised that the methanogenesis stage is particularly sensitive to increased phenol content (Chapleur et al., 2016). The inhibitory effect of phenols is related to their ability to damage the cell membranes. Their toxicity results from both molecular size and non-polarity, as well as substitution in the aromatic ring. They might affect the production of VFA and finally methane (Hernandez and Edyvean, 2008). When analysing the content of both phenols and caffeine in digestate, it might be noted that both compounds were degraded during the AD process, excluding co-digestion of SS and non-cavitated SCG (S1) for which the concentration of phenols increased. This observation indicates the beneficial impact of HC on the degradation of phenols in AcoD.

### Biogas production and its kinetics

**Fig. 2:**
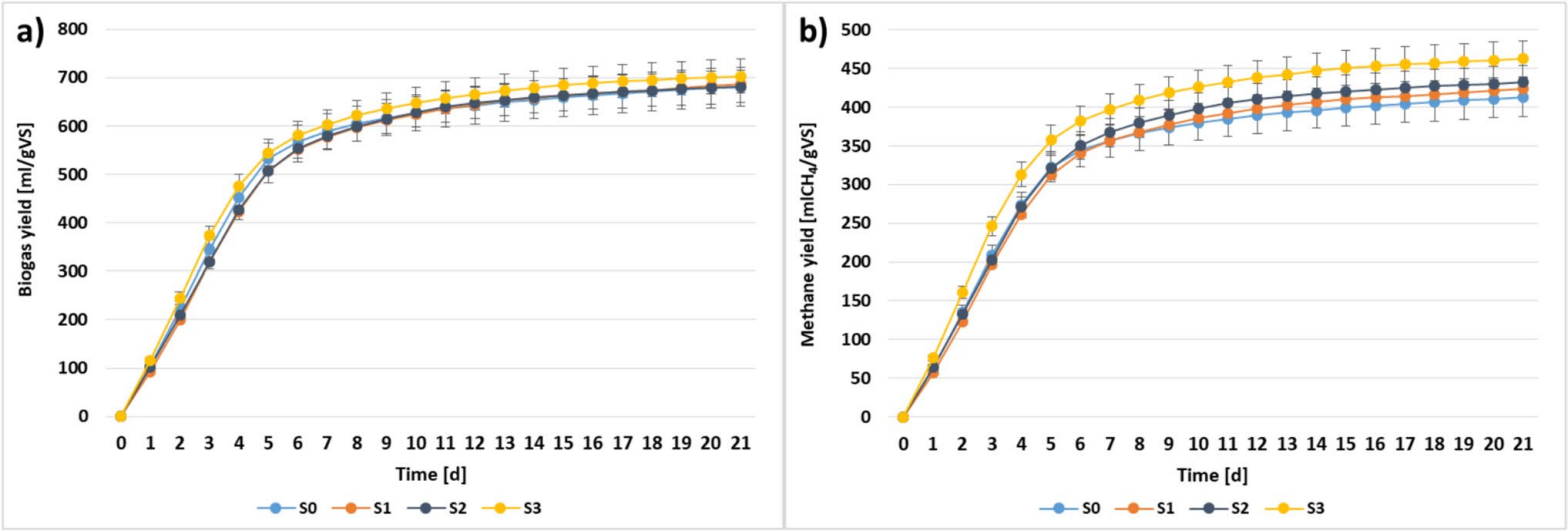
The cumulative biogas (**a**) and methane (**b**) productions in corresponding series (avg. ± SD, *p* < 0.05)

The results in terms of biogas and methane production are presented in Fig. 2 and Table 4. With regard to biogas production, as compared to SS mono-digestion, the increased values were achieved only in S1 and S3; however, they were of no statistical significance. Nevertheless, taking into consideration the implementation of this technology on existing WWTPs, methane production is of the greatest importance. In this case, in all co-digestion series, the increase of methane yield was achieved as compared to SS mono-digestion with the significant growth by 12% in S3 (Table 4), supplied by SCG cavitated for 30 min; importantly, the highest content of caffeine was reported in this reactor. It can be concluded that, in this case, the effect of caffeine was stimulating on methane-producing microorganisms. This fact should be highlighted because the use of co-substrates with a high carbohydrate content often leads to methane content reduction in biogas (Zhang et al. 2022).

In the case of GPR, there was no influence of co-substrate application. In turn, MPR in all co-digestion series was enhanced, the greatest in S3. The observed enhancement in cavitated SCG might be related to significant improvement of its biodegradability related to the destruction of organic matter during HC, which is a result of the impact of both free radicals and shockwaves, as well as thermal effects (Sun et al. 2022). Application of HC allows for breaking the recalcitrant structure of SCG, mainly hemicellulose and lignin, thus providing easy access for enzymes (Bimestre et al., 2022). In both cavitated mixtures, a significant increase of soluble fraction occurred (sCOD) with simultaneous reduction of complex organic matter (Table 2). As it was mentioned before, the major improvement was found in S3 with enhanced caffeine presence. In this study, despite its relatively high content, the influence of caffeine is stimulating. Still, the mechanisms of conversion of this compound are not fully recognised under anaerobic conditions. Some studies indicated that this alkaloid might be used as a source of organic and nutrient nitrogen (Mohapatra et al. 2006). Moreover, Chen et al. (2018) demonstrated that the caffeine-degrading bacteria might be connected with the methane-forming archaea. Accordingly, thorough research in this area should be continued. In particular, the enzyme analysis can indicate the possible metabolic pathways occurring within caffeine biotransformation. Therefore, this research was scheduled as the next step of investigation. Comparing the obtained results with other studies where coffee waste was applied, relatively high values were achieved. Moreover, there is a significant improvement in methane production that is not obtained in many studies. The crucial role in effective AcoD of SCG is the selection of appropriate co-substrate that will promote a synergetic effect and establish adequate process conditions. Ulva biomass is recognised as a suitable co-substrate for SCG that allows for stable AD of SCG. In this case, as compared to SCG mono-digestion, the introduction of Ulva resulted in almost double the increase of methane yield (Kim et al. 2017a, b). In another study, SCG was co-digested with other organic wastes, i.e. Ulva, food waste, whey and waste activated sludge. In this case, the negative effect on methane yield and its production rate was achieved only for waste activated sludge. For other co-substrates, a synergetic effect occurred that resulted in enhanced methane yield. The most beneficial results in methane production were found in the presence of food waste; therein, the methane yield was 355 mLCH_4_/g VS (Kim et al. 2017a, b). In the research conducted by Atelge et al. (2020), the pre-treatment of SCG using oil extraction as well as AcoD with various co-substrates, e.g. tea waste, glycerin, and macroalgae, was evaluated. The highest methane yield was achieved in the case of mono-digestion of pre-treated SCG with the average value of 336 ± 7 mL CH_4_/g VS. In turn, Kampioti and Komilis (2022) co-digested coffee waste with cow manure, food waste and sewage sludge. In this case, the co-digestion with SS resulted in a synergistic effect, with biogas production of 201 mL/g VS; this value was enhanced by 12%, as compared to SS mono-digestion.

**Table 4:** Effect of HC on biogas/methane productions of organic wastes—literature review and results achieved in this study

Feedstock composition	Parameters of HC	Operational parameters of AD	Biogas/methane productions	References
Sugarcane bagasse	Vortex-based hydrodynamic cavitation 9 passes HC	mesophilic conditions (41 °C)	229 mLCH_4_/gVS (increase the methane production by 113%)	Nagarajan and Randade (2022)
Oily wastewater and waste activated sludge	HC retention time of 7.5 min, use of 3-mm-diameter orifice, pressure 6.5 bar	Mesophilic conditions (37 °C)	1089.2 mL/gVS (3.4 times greater than SS mono-digestion) 80% oily wastewater	Habashi et al. (2018)
Agricultural biomasses (pig slurry, maize silage, triticale silage, beet molasses, corn meal)	Rotor HC Inlet pressure of 2.0 bars Specific energy input of 740 kJ/kgTS	Mesophilic conditions	159.1 mLCH_4_/gVS (increase by 14% as compared to un-treated sample)	Garuti et al. (2018)
Food waste and blackwater	Rotor HC	Mesophilic conditions (35 °C)	429 mLCH_4_/gVS Enhanced methane production by 63% as compared to untreated sample	Mahoney et al. (2024)
Food waste and sewage sludge	HC Treatment time of 50 min, pressure of 2.0 bar, applied power of 17 kW	Mesophilic conditions (37 °C)	420 mL/gVS Increased biogas production by 32% as compared to untreated sample	Lanfranch et al. (2022)
Agricultural residues (cattle manure and straw wheat)	HC rotor 4 kW, 2800 rpm	Mesophilic conditions (35 °C)	430 mLCH_4_/gVS Enhanced methane production by 17% as compared to untreated sample	Zieliński et al. (2019)
Maize silage	HC rotor Energy density of 10 kJ/L (291 kJ/kgTS)	Mesophilic conditions (37 °C)	492 mLCH_4_/gVS Enhanced methane production by 34.4% as compared untreated sample	Żubrowska-Sudoł et al. (2020)
Brewery spent grain and sewage sludge	HC orifice plate 3 mm, pressure of 7 bar	Mesophilic conditions (35 °C)	Biogas production 480 mL/gVS Enhanced biogas production by 17% as compared to control Methane production 260 mLCH_4_/gVS enhanced methane production by 13% as compared to control	Szaja et al. (2021)
This study	HC orifice plate 3 mm, pressure of 5 bar, time of 30 min	Mesophilic conditions (37 °C)	462.3 mLCH_4_/gVS Enhanced methane production by 12% as compared to SS mono-digestion and by 9% as compared to untreated sample	-

Hydrodynamic cavitation prior to AS has been applied to various substrates; Table 4 shows the recent studies related to using this method for the pre-treatment of organic wastes. The obtained values of methane/biogas productions depend on the type of applied substrate; the highest value was obtained for oily wastewater. However, HC is mostly applied to lignocellulose biomass. Comparing the obtained results with other lignocellulose materials, comparable results were achieved. Another factor that influenced the HC results is the type of HC device, i.e. rotor, orifice or Venturi, and the adopted operational parameters, i.e. time, pressure and energy.

The results of kinetic analyses are shown in Table 5. Two models were chosen, i.e. logistic growth and modified Gompertz. The high values of *R*^2^, e.g. above 0.99 in all analysed cases, confirmed that these models are suitable for simulating the kinetic parameters of methane generation. However, the RMSE values indicate that among the two models, modified Gompertz demonstrates a better fit.

**Table 5:** The kinetics evaluation with experimental data (average values and standard deviation are given)

Model	Parameters	Unit	S0	S1	S2	S3
Experimental data	BP	mL/gVS	705.7 ± 34.9	725.3 ± 44.3	703.8 ± 22.7	718.4 ± 13.8
	P	mL/g VS	412.6 ± 17.3	423.6 ± 3.6	431.8 ± 7.1	462.3 ± 21.3
	GPR	d	33.60	34.54	33.51	34.21
	MPR	-	19.65	20.17	20.56	22.02
	Methane content		60.59 ± 0.5	61.75 ± 3.4	63.34 ± 1.9	65.76 ± 2.5
Logistic growth model	*P*_m_	mL/gVS	394.6	404.9	415.9	443.3
	*R*_m_	mL/VS d	74.40	69.43	70.70	80.73
	*λ*	d	0.37	0.39	0.32	0.20
	*R*^2^	-	0.99283	0.99273	0.99333	0.99097
	RMSE	%	10.41	10.75	9.18	11.00
Modified Gompertz	*P*_m_	mL/gVS	398.5	409.6	420.5	447.8
	*R*_m_	mL/gVS d	75.01	70.22	71.90	82.27
	*λ*	d	0.23	0.26	0.20	0.10
	*R*^2^	-	0.99712	0.99737	0.99777	0.99636
	RMSE	%	8.11	8.03	6.51	8.38

The results of kinetics evaluation correspond to experimental data, e.g. improved *P*_m_ was achieved in all co-digestion series. However, the major growths by 5.5 and 12% were found in S2 and S3, respectively. The lag phase describes the adaptation time of the microbial community to the new environment. Previous studies indicated that mono-digestion should be characterised by the shortest value of this parameter; in turn, in the presence of a co-substrate, this phase is extended (Farghali et al. 2023). This effect occurred only by using non-cavitated SCG (S1). In the present study, in both series with cavitated SCG, a shortening of *λ* was observed. Importantly, in S3, the greatest values of 46 and 57% were achieved for logistic growth and modified Gompertz models, respectively. Another beneficial effect observed in S3, which is particularly desirable when using lignocellulosic biomass in AcoD, is the acceleration of *R*_m_ by approx. 9% in comparison to control (S0). Conversely, in S1 and S2, this constant was reduced. This constant describes the rate of methane generation and methanogenesis occurring in digesters. This also confirmed the synergistic effect observed in co-digestion of SS and cavitated SCG with enhanced caffeine presence. The improvement of *R*_m_ was also achieved in co-digestion of Ulva and SCG, indicating enhanced microbial activity (Kim et al. 2017a, b).

### Energy balance and economic analysis

The performance of energy balance and economic analysis is particularly important in an industrial application of pre-treatment strategy. HC needs an additional energy input; therefore, it should be evaluated that the obtained surplus of methane production fulfils this energy requirement (Kim et al. 2023). As it is summarised in Table 6, the positive energy gain was found in all AcoD series. Importantly, in the case of HC application as a pre-treatment method, significant energy profits were also achieved; the most beneficial effect occurred in the reactor supplied by cavitated SCG by 30 min (S3). This achievement is of particular importance in the case of full-scale application of this technology. Moreover, it should be pointed out that comparable energy profits were achieved in S1 and S2, therefore excluding the AcoD of SS and cavitated SCG by 20 min for implementation on a technical scale.

**Table 6:** Energy balance and economic analysis in corresponding series

Parameter	unit	S0	S1	S2	R3
Energy balance					
Theoretical thermal energy	MJ/d	58395	61728	60388	64789
Thermal energy for heating the feedstock	MJ/d	14175	14175	13650	13650
Thermal energy for covering the heat loss	MJ/d	3766	3766	3766	3766
Thermal energy demand	MJ/d	18838	18838	18287	18287
Energy demand for HC	MJ/d	-	-	195	294
Total energy demand	MJ/d	18838	18838	18481	18581
Profit of thermal energy	MJ/d	39557	42890	41907	46208
Profit of thermal energy	%	210,0	227,7	226,8	248,7
Net thermal energy profit	%	**-**	17.7	16.8	38.7
Economic analysis					
Energy profit	MWh	11.0	11.9	11.6	12.8
Profit from the sale of biogas	€/a	494457	536130	523835	577604
Transport cost	€/a		39600	39600	39600
Profit	€/a	494457	496530	484235	538004

This finding was also confirmed in the economic analysis. This evaluation was conducted for one digester for which an energy balance was performed. The daily dose of SCG for one digester was established at 469 kg. Therefore, to meet the daily demand for one digester, substrate from approximately 22 cafes should be supplied (assuming that the cafe serves an average of 700 coffees, and each one yields 30 g). The performed estimation proves that the proposed solution is possible to implement on a technical scale. It should be noted that SCG is available all year round, which gives it an advantage over other substrates used in the co-digestion process. Additionally, the location of the cafe in the city allows avoiding significant costs associated with long-distance transport to municipal WWTP. The transport cost was established at 1.1 €/km. It is estimated that the daily route of the transport vehicle will be 100 km (medium-sized city). Therefore, the average transport cost is estimated at 110 €/d. The price for 1 MWh of energy produced from biogas was 125 €/MWh.

As it can be seen in Table 6, only in R3, the profit enhanced by approx. 9% as compared to control (S0) was achieved. The cost of purchasing a cavitator is on average 50,000 €; therefore, its purchase would pay off after 13 months of operation of the installation, contributing to permanent profits for the WWTP.

Żubrowska-Sudoł et al. (2020) applied HC for pre-treatment of maize silage; therein, the positive energy balance was achieved. The related energy profit of 599%, 253%, and 108% was only found for energy densities of 10 kJ/L, 20 kJ/L, and 35 kJ/L, respectively. However, increasing energy resulted in a negative energy balance. Positive effects of HC on energy gains were also observed in a study conducted by Garuti et al. (2018), where HC was applied for agricultural biomasses. In this case, the use of specific energy inputs of 470 kJ/kgTS led to obtaining a stable methane production as well as lower electrical energy consumption by 17%. Zupanc et al. (2023) applied HC for pre-treatment of waste activated sludge. In this case, the negative energy and economic balances were achieved, indicating the necessity for further research in this area. In turn, Son et al. (2025) applied the rotational hydrodynamic cavitation reactor for sludge disintegration; in this case, the positive energy balance was achieved, with a 20% increase in energy efficiency found.

### FT-IR/ATR

**Fig. 3:**
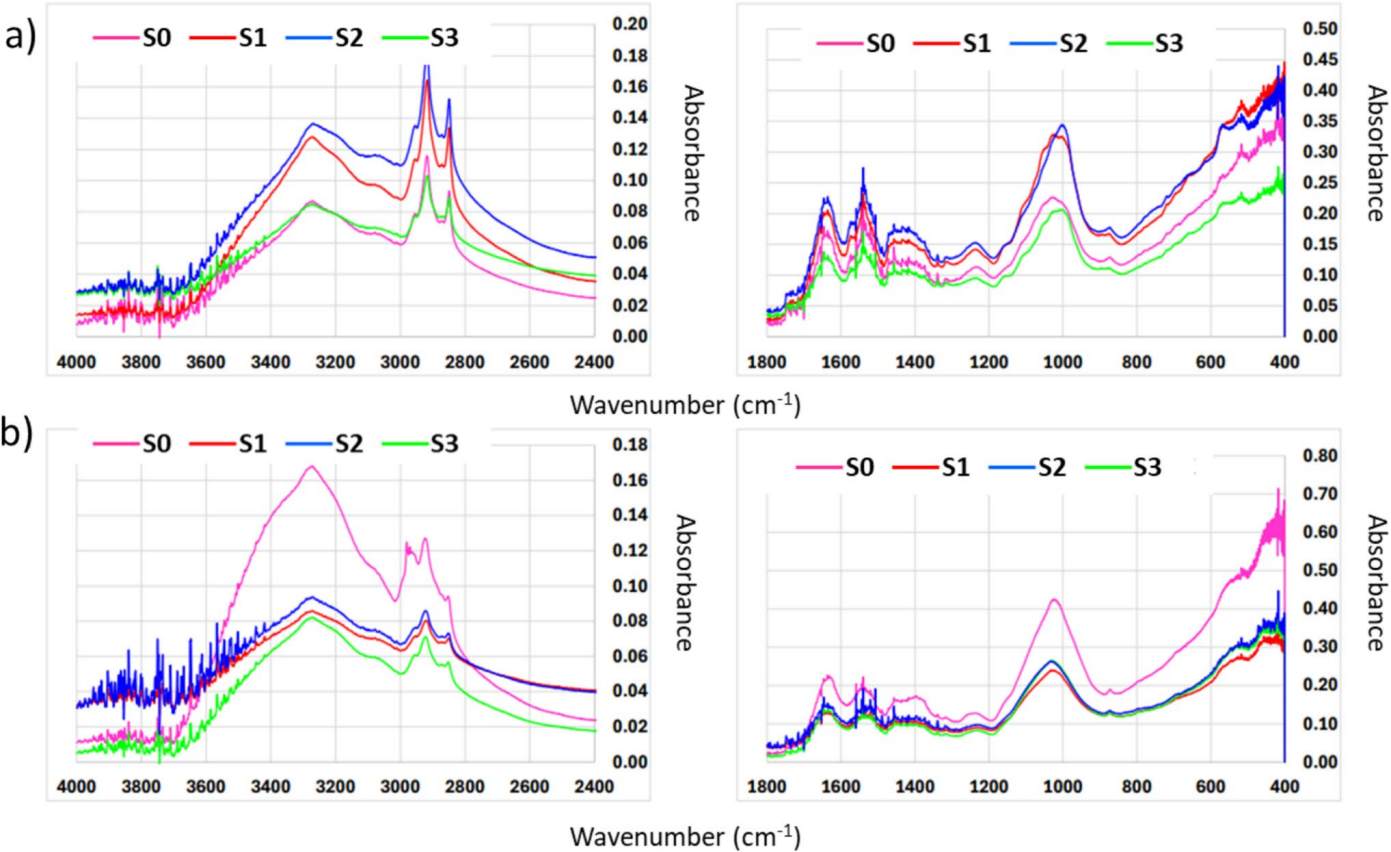
FT-IR/ATR spectra of **a** feedstock and **b** digestate in corresponding series

The FT-IR/ATR method was used to estimate changes in the composition of organic matter occurring within anaerobic digestion. Figure 3 illustrates the spectra of feedstocks (Fig. 3a) and digestates (Fig. 3b) in corresponding series. The analysed spectra constitute a complex mixture of various types of organic substances, i.e. alcohols, polyphenols, fats, fatty acids, proteins, carbohydrates, humic compounds, lipids, lignin, celluloses and various types of difficult-to-biodegrade organic matter. The proportions of these components in the mixture can vary greatly; importantly, the introduction of coffee wastes into the feedstocks resulted in the additional presence of such compounds as caffeine (3,7-dihydroxy-1,3,7,-trimethyl-1H-purine-dione) and organic acids, e.g. chlorogenic acid, coffee acids and lipids (found here mainly in the form of oils and polyphenols). When comparing the spectra before (Fig. 3a) and after (Fig. 3b) the AD process, a decrease in the intensity of the signals might be observed, which confirmed the effective decomposition of organic content within AD. The presence of alcohols and polyphenols in the analysed samples is confirmed by a very broad peak at around 3270 cm^−1^, directly related to OH oscillations, and its width is related to the presence of hydrogen bonds. In feedstocks (Fig. 3a), the intensity of the peak is much higher as compared to digestates (Fig. 3b) also indicating the degradation of these compounds within the AD process. There is a clear difference regarding the spectra of digestates for S0 and other series, indicating much better degradation efficiency in co-digestion experiments, particularly in S3. This is consistent with the results regarding biogas and methane production as well as kinetics.

Additionally, in the presented spectra, very complex structures, bands that are characteristic of chlorogenic acids (around 1380, 1235,1150, 1050 cm^−1^) and esters formed by quinic acid or some trans-cinnamic acids might be noticed. The peaks that appear in the spectral range 1150–1050 cm^−1^ are associated with C-O deformation vibrations, which can be found in coumaric, chlorogenic or coffee acids. The spectrum of pure caffeine contains peaks in the range 2850–2950 cm^−1^, associated with symmetric stretching vibrations of the CH_3_ group and, more specifically, the C-H bond in this methylene group. The peak for the purine ring occurs in the range of 3000–3100 cm^−1^. The peak around 3000–3100 cm^−1^ corresponds to the C-H stretching vibrations that occur in aromatic rings; the bands around 2956 cm^−1^ are vibrations of the methylene group. Vibrations of the C=0 group in the case of caffeine, which does not contain a classical carbonyl group, are much less pronounced peaks occurring in the range of 1650–1750 cm^−1^. Vibrations of the C-N bonds in the purine ring are peaks in the range of 1200–1350 cm^−1^, while the C-C vibrations that occur in the purine ring are peaks in the range of 1000–1600 cm^−1^. The vibrations of the CH_3_ group located at the nitrogen atoms for positions 1, 3, and 7 in the range 1380–1450 cm^−1^ might be also observed. When analysing the spectrum of caffeine, these peaks can vary depending on the concentration state of the molecule (pure state or mixture). In the case of the analysed samples with a complex composition, the peaks of caffeine overlapped with the bands of lipids, polysaccharides or proteins, which makes it very difficult to accurately interpret in such a multifaceted system. Despite this fact, FT-IR/ATR analysis of even very complex mixtures can provide useful information about the chemical structure of the materials studied and estimate the degree of distribution of functional groups. The smallest differences in the spectra can be observed for mono-digestion of SS. It should be emphasised that, despite the complexity of the mixture, the bands of caffeine and acids can to some extent overlap with peaks from other components of the analysed medium.

### SEM analyses

**Fig. 4:**
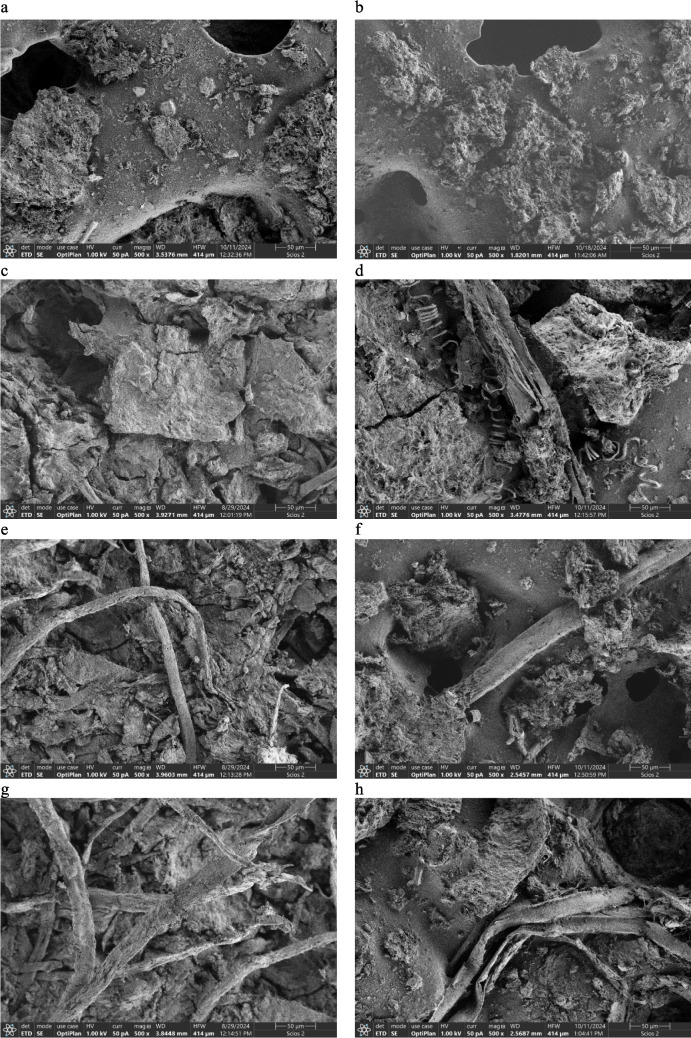
SEM images of **a** S0 feedstock, **b** S0 digestate, **c** S1 feedstock, **d** S1 digestate, **e** S2 feedstock, **f** S2 digestate, **g** S3 feedstock, and **h** S3 digestate

The microstructural changes of samples before and after anaerobic digestion are presented in Fig. 4. The most homogeneous structure was obtained both in F and D of S0, in which mono-digestion of SS was conducted. The introduction of co-substrate resulted in changed structure of the feedstocks. In these cases, the heterogeneous structure of pores and rough texture might be observed. However, the most visible changes were found in both cavitated samples (S2 and S3) (Fig. 4e,g). In particular, in S3 where the highest cavitation effect occurred, significant fragmentation of the lignocellulosic structure was achieved due to the prolonged HC duration. This fact resulted in increasing the digestibility of lignocellulosic fibres and hence significantly improved biogas and methane productions (Table 3). Importantly, the previous studies also confirmed a high efficiency in disruption of lignocellulosic biomass using HC (Sun et al. 2020). This phenomenon is accompanied by extraordinary conditions, such as high pressure, local hotspots and generation of hydroxyl radicals. Additionally, within HC, the shock waves and shear stresses demonstrate the potential to break down the complex lignin-carbohydrate matrix in the biomass, making the cellulose and hemicellulose more accessible for further anaerobic conversion (Bimestre et al. 2022).

In the entire digestate, as compared to feedstock images, the structure was more homogenous, resulting from the degradation of organic matter within AD. The lignocellulose fibres might still be visible; nevertheless, their structure is highly disturbed. Interestingly, in non-cavitated SCG digestate, the structure was visible as more heterogeneous; Fig. 4d shows numerous non-decomposed fibres of SCG.

## Implications and limitations

This study presented a technology that includes a combined method including pre-treatment using HC and subsequently anaerobic co-digestion of pre-treated SCG with SS. Applying such a strategy always brings technological and economic challenges. It should be highlighted that the results presented in this research are preliminary; therefore, it should be continued in semi-industrial scale at existing WWTPs. Implementation of this strategy at WWTPs is related to additional financial outlays related to the purchase of a cavitator and additional equipment necessary to connect the device to the working digesters. It should be mentioned here that the economic analysis performed showed a quick return on investment. Additionally, it requires training of employees who will use the device. During technological start-up, numerous digester-related problems may arise, e.g. foaming. Occasionally, it may be necessary to adjust the operating parameters of the cavitator. Another challenge might be combined with ensuring a continuous supply chain of SCG. However, it should be noticed that this waste is widely available all year round. Another important issue that is often overlooked is the social and environmental factor. This technology allows limiting the negative impact of this waste on the environment and also allows exploiting the potential of SCG generally considered to be an unsuitable material with simultaneous use maximisation of existing digesters that are often underloaded. This might be achieved by popularisation of science in public opinion.

## Conclusion

In this research, a novel strategy for improving the energy balance of WWTP using cavitated SCG was proposed. As compared to SS mono-digestion, a statistically significant growth in methane yield was achieved in the presence of SCG cavitated for 30 min with the highest caffeine content. Importantly, the improved methane production rate by 9% and a major shortening of the lag phase occurred. The results obtained with regard to methane production contributed to the achievement of significant energy gains of 39%, as compared to SS mono-digestion. Additionally, the results of economics indicated that in the case of SCG cavitated for 30 min, a profit was enhanced by approx. 9% as compared to SS mono-digestion. All the mentioned achievements resulted from the destruction of the recalcitrant structure of SCG that occurred within HC and simultaneous biodegradability improvement. Moreover, in this case, the stimulatory effect of caffeine might be observed. The obtained results are of particular importance due to the growing energy crisis and the depletion of non-renewable energy sources. The proposed technology allows for the successful management of SCG with simultaneous energy recovery; moreover, it might contribute to achieving energy neutrality of many WWTPs.

## Data Availability

The datasets used and/or analysed during the current study are available from the corresponding author on reasonable request.
